# Supplementary material for: 1-Deoxysphingolipids Require Very-Long-Chain Ceramide Synthesis to Induce ER Stress and Neurotoxicity
Source: bioRxiv. 2025 Dec 8:2025.12.06.692689. Preprint. [Version 1] doi: 10.64898/2025.12.06.692689 (PMC12710655; doi:10.64898/2025.12.06.692689)
Supplement: Supplement 1 [file media-1.pdf]

Supplemental Information:

Table S1. Top 30 positively ranked genes derived from 1-deoxysphinganine resistance screen in SH-SY5Y cells analyzed by Model-based Analysis of Genome-wide CRISPR/Cas9 KO (MAGeCK).

id	num	pos|score	pos|rank	pos|lfc
TECR	6	9.49E-18	1	6.7474
ACACA	6	1.28E-11	2	3.4526
CERS2	5	7.78E-11	3	3.0367
PTPLB	4	2.18E-09	4	3.7038
HSD17B12	5	2.70E-07	5	0.90363
hsa-mir-5698	1	3.38E-05	6	12.358
ACLY	2	0.00060904	7	2.1624
HLCS	3	0.00069476	8	0.90363
hsa-mir-3609	3	0.00069476	9	0.90363
ELOVL1	3	0.00069476	10	0.90363
NDUFS8	1	0.00071066	11	5.8485
SRGAP1	1	0.0010491	12	5.0126
SLC46A2	1	0.0011168	13	4.9548
hsa-mir-4278	1	0.0011844	14	4.8318
FAM3D	4	0.0011966	15	-0.72803
hsa-mir-3677	1	0.0013875	16	4.6916
PSPH	1	0.0015567	17	4.2935
DHRS7C	2	0.0016237	18	0.42207
WBP5	1	0.0017259	19	4.1971
CCDC101	1	0.0018274	20	4.0938
KAAG1	1	0.0020981	21	3.7305
LHX8	1	0.0020981	22	3.7305
SLC2A11	1	0.0020981	23	3.7305
PLA2G3	2	0.0026379	24	1.9105
RNF144A	1	0.0026734	25	3.4249
FGF16	3	0.0029413	26	-2.9191
DYNLL2	1	0.0031472	27	3.2438
KLF1	1	0.0031472	28	3.2438
TACSTD2	1	0.0031472	29	3.2438
SSUH2	1	0.0031472	30	3.2438
